# Carbon Accounting in the Digital Industry: The Need to Move towards Decision Making in Uncertainty

**DOI:** 10.3390/su16052017

**Published:** 2024-02-29

**Authors:** Gabrielle Samuel, Federica Lucivero, Bran Knowles, Katherine Wright

**Affiliations:** 1 Department of Global Health and Social Medicine, King’s College London, Strand, London WC2B 4BG, UK; 2 Ethox Centre, Nuffield Department of Population Health, University of Oxford, Old Road Campus, Oxford OX3 7LF, UK; 3 School of Computing and Communications, Lancaster University, Lancaster LA1 4WA, UK

**Keywords:** carbon emissions, sociology of knowledge, digital technologies, ICT, uncertainty, adaptive governance

## Abstract

In this paper, we present findings from a qualitative interview study, which highlights the difficulties and challenges with quantifying carbon emissions and discusses how to move productively through these challenges by drawing insights from studies of deep uncertainty. Our research study focuses on the digital sector and was governed by the following research question: how do practitioners researching, working, or immersed in the broad area of sustainable digitisation (researchers, industry, NGOs, and policy representatives) understand and engage with quantifying carbon? Our findings show how stakeholders struggled to measure carbon emissions across complex systems, the lack of standardisation to assist with this, and how these challenges led stakeholders to call for more data to address this uncertainty. We argue that these calls for more data obscure the fact that there will always be uncertainty, and that we must learn to govern from within it.

## 1 Introduction

Climate change is one of the most pressing challenges currently facing society. International and national attempts to help mitigate climate change have focused on accountability practices that require the collection of data to assess carbon emissions, to set goals, and then gauge progress towards these goals [1]. This has meant that climate change has become what scholars have described as ‘*a problem of gathering data and acting on that data within the terms set by these modes of calculation*’ [2]. This approach is now so central to climate change policies that such policies both presume and require the systematic quantification of carbon emissions [3]. Implicit in this is the assumption that if we can make carbon emissions visible and accountable through quantification, we can better understand and take steps to reduce them [4]. This has reinforced modernist assumptions that place faith in the ability to solve climate change challenges through calculating and then managing carbon emissions [5,6]. As such, we have seen a range of carbon accounting tools and burgeoning guidelines and frameworks to help support individuals, businesses, institutions, and nations calculate carbon emissions (for example, see [7–9]).

At the same time, carbon quantification has a range of difficulties and associated challenges, meaning that it often provides uncertain answers. While in the broader literature, responses to uncertainty often call for more data (for example, see [10] for discussion); as we continue to highlight, carbon quantification is not a fact-finding mission to some ultimate certain truth, nor is there a ‘correct’ scientific way [11] to quantify and calculate carbon emissions; moreover, calculating carbon does not represent the amount of material carbon ‘out there’. Rather, carbon calculations are just representations of the world [12,13], which are constructed through socio-technical processes, relationships, and interactions between actors, organisations, information, and policies [1,2,5,14]. This means that if we want to engage with concepts of uncertainty and certainty, we need to better understand these socio-technical systems.

In this paper, we explore the socio-technical processes of carbon accounting in the digital sector, and, through doing so, call into question appeals for more data to address the uncertainty associated with quantifying carbon emissions. To do this, we draw on a qualitative interview study designed to explore stakeholder views, experiences, and practices in the field of sustainable digitisation. While interviewees were working in different areas of sustainable digitisation, many participants were engaged in carbon accounting and/or used carbon accounting data in their practices: this paper reports on this specific topic that emerged in the interviews. Building on a critical realist approach, we view knowledge, understanding, and beliefs associated with carbon accounting as socially constructed; at the same time, we acknowledge that a reality exists independent of these social constructions [15]. Our research question was as follows: how do practitioners researching, working, or immersed in the broad area of sustainable digitisation (researchers, industry, NGOs, and policy representatives) understand and engage with quantifying carbon? Our findings show how these stakeholders struggled to measure carbon emissions across complex social and political systems, and the lack of standardisation to assist with this. As our participants tried to move towards a state of certainty associated with carbon accounting so that standards could be implemented, they were hindered by a range of social and political challenges, such that uncertainty remained. In the discussion, we argue that without engaging with these insights about carbon quantification, similar to our interviewees, there is a greater tendency to think that current uncertainty in carbon accounting can be addressed with evermore data accumulation. We emphasise the infeasibility of attaining certainty through this data collection and the need to accept uncertainty, take action despite it, and govern within it [16]. We introduce insights from studies of uncertainty as a means of moving productively through the challenges experienced by our participants and call for more research to better understand what such an approach would look like in the digital sector. Before doing so, we provide a brief overview of carbon quantification, introduce our case study on digital technologies, and then move on to our methodology and findings.

## 2 A Brief Overview of Quantifying Carbon

Carbon emissions are typically classified according to the greenhouse gas protocol, which divides emissions into three scopes. Scope 1 and 2 are associated with the *operational aspects* (or ‘*use*’ *phase*) of a particular innovation, organisation, or sector. Specifically, scope 1 comprises direct carbon emissions produced by an innovation, organisation, or sector; scope 2 consists of indirect emissions produced by third parties to provide electricity to power an innovation, organisation, or sector. Scope 3 encompasses emissions resulting from the *upstream* manufacture and production of a product (i.e., the ‘embodied emissions’); other indirect emissions, such as those associated with employees commuting to work; and the *downstream* emissions associated with the use of a product or the product’s end of life related processes. Different methodologies, calculators, and frameworks have been developed to assist with carbon accounting. Life cycle assessments (LCAs) are the more notable for including scope 3 emissions. These measure carbon emissions across the life cycle of a product, organisation, or sector (LCAs may assess many environmental impacts associated with a product’s, organisations, or sector’s lifecycle, but here we focus on carbon emissions), and are often called a cradle-to-grave analysis. LCAs require considerable time and judgment, are plagued by issues of uncertainty and lack of data associated with upstream and downstream activities, and, as such, they are the hardest to calculate. Furthermore, they are associated with a range of divergent methods. Nevertheless, the importance of LCAs is increasingly being recognised; nowadays, evermore analyses are being published in this area and/or being placed on various databases for others to access and use in their own calculations (for example, see [17]). (Also, see [18]). Furthermore, companies are investing in sustainability experts and/or using sustainability frameworks to help them navigate these assessments and champion decision making based on the findings [19].

## 3 Digital Technologies

Digital technologies (DTs) allow for the datafication of things; including the gathering, storing, and processing of data for various uses, including machine learning technologies and other artificial intelligence (AI) algorithms. Examples of DTs include data centres; information and communication technologies (ICTs); the internet of things (IoT); and digital infrastructures and devices. Moreover, digital technologies (DTs) have an important role to play in mitigating climate change. For example, DTs are often viewed as a driver for reducing the carbon emissions of various sectors by providing information to reduce energy consumption [20,21]. DTs can also be used for the analysis of very diverse datasets and for supporting climate related decision making when dealing with highly complex environmental systems (for example, see [18Lam, 2023 #2614]). The efficiencies DTs deliver promise to be harnessed across the economy to enable the continuance of a comfortable way of life as society downshifts carbon emissions [22]. Despite this exciting potential, there is growing concern regarding the carbon emissions of DTs themselves. These arise during the manufacture of devices and device components, the use of DTs (e.g., the storage and processing of large amounts of data), and the disposal of hardware [23]. Global carbon emissions of DTs are, according to some experts, steadily rising and will continue to rise despite continual improvements in efficiency [23]. This is because, while likely improvements in energy efficiency and the move to renewable energy relieves at least some of these concerns, the pace of digital innovation could outpace the world’s renewable energy sources, leading to increases in carbon emissions when other sectors are decreasing their energy use [23,24]. Furthermore, the rebound effects of DT solutions mean that while increases in energy efficiency may be perceived to offer environmental advantages, they may likely lead to an increase in digital consumption [25–27]. As such, the sector is now facing pressure (like all other sectors) to find ways to reduce its emissions through carbon accounting.

This growing concern has led to a strong focus on the measurement of carbon emissions associated with DTs. A range of tools have been developed to help calculate the use phase (phase 1 and 2) emissions related to DTs, and many groups are producing guidance and tools to help with the carbon accounting of digital products, practices, and processes (for example, see [28]). This includes, for example, carbon calculators and/or frameworks to assess emissions associated with software; machine learning and other artificial intelligence algorithms; and/or other digital processes [29–31] (also see [32,33]). At the same time, compared to other industries, there is still little regulatory pressure to quantify emissions in the digital sector because of an oftentimes lack of requirements for formal approvals for digital sector expansion compared to the need for other sectors to report on any potential environmental impacts that may be associated with their activities, for instance, in the construction industry. This is perhaps compounded by the fact that the digital sector is often seen as a technological solution to reducing emissions produced by other sectors and/or because, in these sectors, digital-related carbon emissions are viewed as less significant than other impacts. (https://greenio.gaelduez.com/e/4n9x9528-31-estimating-it-footprint-the-abb-case-study-with-fiona-leibundgut-and-thomas-mosser?utm_source=climateActionTech&utm_medium=email&utm_campaign=cat-newsletter-206-2024-02-11) (accessed on 21 February 2024). Most of the developed tools for measuring carbon emissions for a digital product, practice, or process do not calculate scope 3 emissions. This is because of the associated difficulties with these processes, which are especially complex in this sector due to the complicated infrastructure of devices, technologies, systems, networks, and digital devices that are manufactured using complex processes with a vast number of different minerals, metals, and other substances. Furthermore, while progress is being made to develop methodologies for calculating the scope 3 carbon emissions associated with DTs, we are still seeing vast discrepancies and disagreement in published quantifications and uncertainties abound in attempting to measure net carbon impacts [23]. The digital sector is pushing for more data to address these uncertainties; however, it is unclear how much certainty in these measurements is needed before action is taken to reduce the DT sector’s carbon emissions. It is this aspect that is the focus of this paper.

## 4 Methods

### 4.1 Interviews

Interviews were conducted in the summer of 2021. Participants were identified from (a) the literature (see below) and (b) snowballing via key stakeholders in the field known to the authors and/other interviewees (at the end of interviews, interviewees were asked for recommendations of other relevant stakeholders to invite to this study). Seventy-three individuals were asked to participate via email and twenty-four individuals accepted the invitation. Table 1 reports the self-reported sector of the interviewees. Interviewees were based in the UK and continental Europe (*n* = 20), continental North America (*n* = 3, and Australia (*n* = 1). Seventeen interviewees were male and seven were female. Interviews were held online or over the phone and broadly explored participants’ roles and work practices as well as their perceived challenges and issues in the digital technology sector as they pertained to environmental sustainability (see Supplementary Materials). For academic experts, this involved asking about their research area(s) and specific disciplinary expertise; the aim of their discipline(s) (what type of knowledge it aimed to uncover); who they engaged with/who engaged with their research; the methodological challenges they faced in their research; and how they viewed their research in relation to sustainability. Analogous questions were asked to non-academic stakeholders, including their professional background, current role, and day-to-day duties; the types of data, knowledge, and stakeholders they engage with in their role; challenges in their role; and how their role intersects aspects of sustainability. All interviewees were questioned about key stakeholders in the field, the main issues in digital sustainability, and what they perceived as necessary to overcome these challenges.

Interview data were thematically analysed [34] using NVivo software 14. GS and a research assistant independently read and re-read each interview transcript and noted down key themes on a memo. A meeting was held to discuss relevant themes and overlaps. GS and the research assistant then independently conducted broad coding of the transcripts for the relevant themes using their own versions of NVivo (for this paper, the relevant theme was related to carbon accounting). The two coding schedules were then merged, and GS then conducted line-by-line coding of the carbon accounting merged code. GS then analysed this coding to draw out key points. During analysis, if sector-specific perspectives were noted, these are reported in the findings.

### 4.2 Identification of Interviewees from the Literature

Alongside snowballing, participants were also identified using a bibliometric approach because it was considered that selecting interviewees in this way would allow us to speak to those who hold the most knowledge and expertise in the field of digital sustainability (in terms of academic research). Articles in Web of Science, published between 2016 and 2021, were searched using four separate keyword string combinations that were developed deductively and inductively (Supplementary Materials: Table S1). The combination of all keyword strings returned 4598 articles. Titles and abstracts of articles were reviewed for relevance. Duplicate reviewing was applied initially to 300 articles to ensure consistency of approach. Inclusion criteria included articles exploring or discussing the environmental impacts of DTs. Articles that discussed environmental impacts that were not specifically associated with the digital aspects of DTs were excluded. A research assistant applied the inclusion/exclusion criterion to the remaining articles. In total, 489 articles remained after review. All publications (*n* = 489) were imported into VOSViewer 1.6.19 (https://www.vosviewer.com/) (accessed on 2 February 2021). VOSViewer collects and organises aggregated articles and allows for the construction of co-authorship relations’ networks of all contributing authors (*forthcoming*); it also allows for the generation of lists of authors according to specified criteria. Lists were generated according to authors who were most represented in our sample in terms of number of publications, as well as those who were most cited. The top 20 authors in each of these lists were invited to an interview based on contact details provided in one or more of their publications.

## 5 Limitations

This was a qualitative exploratory study: our aim was not to speak to a large, representative sample of individuals, but rather to purposively sample those individuals working across the field of digital sustainability, with the goal of inductively analysing this smaller dataset—in-depth—to generate hypotheses. While individuals from a range of professional roles were represented in our sample, this hypothesis-generating approach means that our findings require testing and, as such, should be open to further scrutiny. Furthermore, our sample of 24 participants was limited. First, there was a lack of low- and middle-income country representations; second, it was predominantly male; and third, it was mainly from Europe and the UK. Our bibliometric findings suggested that, to a degree, these limitations were perhaps because of the gender gap in the field, as well as the low representation of low- and middle-income countries in the literature. It is essential that further research works towards ensuring that the voices of experts who are working in the space of carbon accounting in the DT sector, but who may not be the most prevalent or well-cited, are also heard.

### Ethics

This study received ethical approval from the Oxford University Central University Research Ethics Committee (CUREC) (reference code: R75723/RE001).

## 6 Findings

Early in the interviews, it became clear that our participants had different views about how much the digital sector contributed to global carbon emissions. Some interviewees worried about what they perceived to be the increasing growth curve of predicted digital technology energy use and the likely increases in data centre energy consumption over time (interviewee 4, business representative; interviewee 16, computer science). Other interviewees argued that their own reported analyses suggested that such concerns were ‘*disproportionate*’, and that ‘*the impacts [of the sector] are relatively small*’ (interviewee 10, digital energy analyst). During the interviews, it became evident that there was a range of uncertainties and challenges associated with quantifying carbon emissions that explained these different views. These were related to difficulties of gaining data about complex systems; the fragmentation of different knowledge communities in the field; and a lack of transparency and standards in the field. These challenges led to gaps in emissions data, incorrect assumptions about data; and personal decision making about how to address these issues. These are described in more detail below.

### 6.1 How Do You Know What to Measure?

This difficulty is associated with either a lack of data altogether, or with a lack of updated data. Furthermore, even when data were present, they were not always deemed usable because the speed with which hardware is often replaced in data centres meant that calculations quickly became outdated, especially with the time lag of peer review publishing. These issues were compounded by a perceived reluctance by industries to release propriety information about their carbon emissions: ‘*it was impossible to get hold of any information on that [for their calculations related to data centres]*… *Even with people we know*… *it was just impossible*’ (interviewee 11, researcher). These more political and economic issues around openness and transparency, which were tied to industry concerns about competition, public image, and trust, affected researchers’ abilities to make accurate predictions of carbon consumption: ‘*with digital in general, I think the main thing is being transparent*… *digital trust and responsibility*’ (interviewee 22, NGO). Interviewee 1, who worked at a large digital company, spoke about their frequent experience of only being provided with such information if they signed a contract that prohibited them from publishing the data externally: ‘*if we ask our suppliers*… *they say, “Yeah, you can use the data, but you can never publish our data externally. You can aggregate it in a product*… *but you cannot sort of talk about our data*”‘. The unwillingness of data centres and/or other industries to disclose information about carbon emissions and/or the lack of information about what these emissions were, meant that there were many gaps in their datasets: ‘*we are having really gaps in the calculations*… *sometimes we see the carbon footprint only for the use phase*… *because they have to go to their own suppliers and accounting is really difficult*’ (interviewee 18, industry).

Moreover, our interviewees explained that this difficulty is especially the case when accounting involves assessing emissions across digital networks and within data centres because the data associated with a particular device, such as a mobile phone, is difficult to entangle from other data present in data centres. This is because data for specific purposes are not contained or constrained away from other data uses: ‘*when you get into the network and the data centre, these are opaque systems*… *it*’*s not possible to detect what device is actually being used, or what devices are running, or what equipment is running on the network or in the data centre*’ (interviewee 6, researcher). Disentangling the emissions associated with the use of one device or service from another is difficult because data centres are ‘opaque’ and entangled networks. The data are not just inaccessible but are also hard to produce, given the infrastructure.

Accounting for carbon emissions when *using* a device or service is only one stage of a comprehensive calculation, which should also include embodied emissions associated with the device or underlying physical infrastructure. There was a perceived lack of data associated with the embodied carbon emissions related to the manufacturing and transport of each component comprising a digital technology, which made it challenging to conduct calculations (interviewee 18, industry). When calculating embodied emissions, one of the most pressing issues was the difficulty in making calculations with all the different data that would be needed if the manufacturing of each component constituting the device had to be considered. Interviewee 11, a researcher, exemplified the issue using a pair of glasses. This interviewee described the various factors a researcher would need to think about when calculating embodied emissions of these glasses, explaining how this would be vastly more complex for digital technologies, which have infinitely more components:

*‘When you start to break down where this pair of glasses comes from in terms of materials, you end up normally with at least*… *200 sources of*… *raw materials*… *which have embodied impact in terms of extracting the minerals, or the raw material, transport, and manufacturing in different parts of the world and comes here [sic].’*

Moreover, interviewees explained how the further upstream a researcher travels to assess embodied emissions, the harder it becomes to decipher how many of the upstream carbon emissions are associated with a specific downstream device. This is because each device component is one very small percentage of an upstream process that has provided components to multiple devices (‘*the further upstream you go the less determinate it becomes in terms of being attributable to electronics, so that*’*s part of the, the complication*’) (interviewee 17, social scientist).

As in the case of disentangling the emissions associated for the use of one device in a data centre, in the case of manufacturing, the issue pertains to the challenge of following the material infrastructures, discerning all its relevant parts, and calculating the emissions for each one of them. Both the intricate nature of data centres’ networks and the multiplicity of components resonate with the idea that these are complex systems.

Even when embodied emissions were included, researchers would make different decisions about what emissions could/should be included in the assessment, and interviewees pointed to how life cycle assessments could be conducted in many ways. This was because decisions needed to be made about where to draw boundaries—the wider you go, the more indeterminate and uncertain the figures; the narrower you go, the less chance of capturing all emissions in the calculations: ‘*there*’*s a key difference in system boundaries [and in]*… *deciding what is going to be measured*’ (interviewee 6); ‘*where do you set the boundary?*… *where do you stop?*’ (interviewee 3, science organisation). With different ways of conducting a life cycle assessment, and with little understanding about what the ‘correct’ [11] outcome should be, disagreements about the most appropriate approach to conducting an assessment were common. In one example, interviewee 19, a representative of a standard bearer in the sector, was concerned about too wide boundary drawing, which meant that researchers bring uncertain figures, and therefore assumptions, into their calculations:

*‘People draw boundaries too big. You’re building boundaries into an area where you have no certainty. So I can make up*… *I can give you a Scope 3 inventory number for an operation but I’ll tell you flat out it’s full of crap. There’s four or five categories where I can give you a good number and then there’s ten categories, right, I mean*… *I’m making stuff up, I’m doing it intelligently but I’m using formulas and options and there’s huge uncertainty to it.’*

The uncertainty was associated with disagreements regarding the relevant data and the methods of calculating emissions which, as discussed below, varied depending on the different research community involved in the assessments.

### 6.2 Different Bodies of Research That Are Looking at Very Different Things

Interviewees recognised that because of the challenges associated with quantifying carbon emissions, to accurately conduct a carbon emissions analysis, understanding and expertise across the whole digital sector were required—not only across supply chains and devices but also across data and energy infrastructures and digital (information technology (IT), Internet of Things) networks. Interviewee 8 remarked: ‘*it*’*s not obvious how the systems behave and the experts are not necessarily experts in network technology*’. Some participants—both academic researchers as well as those in industry—explained how they recognised this and stated that collaborations were part of their everyday practices: ‘*I collaborate with multiple people*… *from electrical engineering, people from mechanical engineering*’ (interviewee 16, academic researcher, computer science) and ‘*we have a specific programme ongoing with 1000 of our suppliers where we innovate together*’ (interviewee 18, industry).

However, during interviews, it was unfolded that many actors in the digital sector were perceived not to be collaborating. Interviewees viewed the sector as comprised of a range of communities of knowledge generation, each trying to achieve the same goal but drawing on different disciplinary methods, literature, and analyses: ‘*different academics are approaching this in different ways*…*[*…*]*… *when people are using the literature, they are often choosing*…*different bodies of research that are looking at very different things*’ (interviewee 6). This meant that each discipline was quantifying carbon emissions in their own way and using their own processes, with disciplinary differences, leading to different ways of viewing the numbers in carbon quantification. Interviewee 6, a researcher, provided the example of ‘top-down’ and ‘bottom-up’ approaches to exemplify how researchers’ different methodologies relied on assumptions from different bodies of research knowledge, which ended up producing different energy consumption figures:

*‘“How much energy does a data centre use?” You’re going to get estimates*… *maybe 4 or 500 terawatt hours of electricity*… *extreme estimates that say 2 or 3000 terawatt hours*… *There’s a lack of consistency in the methodologies*…*[*…*]*… *There’s a top-down number where you say, “Well, what is the total energy used by IT in a particular region? And how many people are there?” And then you just divide those two numbers*… *Or you can do what’s called a “bottom-up approach”, where you calculate the energy consumption of each individual piece of equipment, and*… *then*… *use that to calculate the energy intensity figure. And they often come to quite different results.’*

Similarly, interviewee 8, an academic in sustainability and computing, used the example of video streaming to explain how those in a different field approached quantification in various ways, meaning that diverse answers were constructed. In the extract below, this interviewee was frustrated with environmental assessment experts, who they perceived to not be up to speed with the latest progress in carbon accounting methodologies for digital technologies:

*‘If you’re an environmental assessment person you look at the network and you make assumptions of that*… *For digital, and we really only started to understand this in the last couple of years*… *we [use a different quantification model] and we began to realise how big the difference between the two were.’*

Interviewee 19 articulated the disciplinary power struggles at play between different research communities when quantifying carbon: because multiple realties can be produced from any set of numbers depending on the way in which numbers are analysed, each discipline tried to push their specific view of how best to quantify carbon emissions:

*‘Because everybody plays games, right, it’s all for discipline and presentation and I like to say that, “Give me a set of facts, then I can create you multiple realities brought across the whole spectrum of approaches.”‘*

Furthermore, interviewees stressed how these communities were fragmented and siloed from one and another; individuals in each community did not communicate in terms of their carbon quantification practices: ‘*the sector has*… *evolved*… *this*… *silo [of] subsectors*…. *Mono-discipline culture is absolutely pervasive in the sector*’ (interviewee 7, academic researcher, design/sustainability) and *‘one of the problems we have with this industry is that it*’*s very siloed, so there isn*’*t a good deal of cross fertilisation [of knowledge]*’ (interviewee 9, scientific lead at a data centre).

This fragmentation of communities was problematic because different communities in the sector (e.g., data centre operators, IT experts, thermal engineers) were each working towards their own goals on joint initiatives while failing to take a more holistic ‘*bigger picture approach*’: ‘*[when] you understand your own discipline, you don*’*t really understand the effects that you have on the other aspects of operation*’ (interviewee 9, scientific lead at a data centre). Although addressing this was perceived to be difficult in practice, ‘*it*’*s almost as though people have to learn how to talk to people from other disciplines, you know, find a common language*’ (interviewee 7, academic researcher, design/sustainability). This worried several interviewees, who were concerned that this made it particularly difficult for researchers to draw on each other’s work across research communities in an appropriate way. The concern, explained interviewee 10, a digital energy analyst, was that when researchers from one community of knowledge draw on data from another, this leads to inaccuracies in modelling. This is because the underlying reliability and/or predictability of the data was black-boxed [35]. This meant that the researchers performing the modelling calculations incorporated this knowledge into their calculations without understanding the underlying methodological assumptions and uncertainties associated with the knowledge, inevitably leading to inaccurate assessments. This interviewee provided an example of researchers fundamentally misunderstanding the relationship between energy and digital devices:

*‘Most internet infrastructure at the data centres, all the data networks, your router at home, they all operate with a very high fixed energy cost, so there is a fundamental misunderstanding by some of these researchers about how the equipment actually operates in reality.’*

Misunderstandings across research communities, differences in methodologies, and a lack of common language brought disagreements among experts. As we see below, such disagreements were not just across disciplines but also within them, and that this, coupled with a lack of standards, made it hard to find a sense of direction in this field.

### 6.3 The Need for a Common Accepted Benchmark

Nearly all interviewees pointed to a lack of sector standards, which raised challenges concerning interoperability and frustrated interviewees because it meant that researchers were using different metrics in their calculations and comparison was difficult: ‘*how it [an organisation] calculates its carbon footprint is possibly not identical to the way that [another one does]*… *there*’*s no way of comparing one to another because they*’*re not operating across the same metrics*’ (interviewee 4, business representative). The lack of standards, as well as the challenges and uncertainties inherent in calculations meant that even within the same research communities (as well as between), interviewees were making their own decisions about which methods were the most appropriate to assess carbon emissions, what to count, and what to leave out of their carbon emission assessments. In the extract below, interviewee 11, an academic researcher working with data centres, describes their own attempts to assign metrics and models to a specific carbon emission problem, all the while realising that because of the heterogeneity of methodologies and metrics used in the field, their metrics will likely be met with more disagreement than agreement, with other researchers preferring to use their own methodologies and calculations:

*‘There’s not an easy metric you can go back to*…*[*…*]*… *just using data from life cycle assessments, we tried to give it some different values here and there. But you know, you’ll probably find more people disagreeing with those values, than agreeing with them*… *and you couldn’t scientifically prove that that was, you know, the right answer, there would be a debate about each, every single one of those values.’*

These differences between carbon accounting methods mattered. Without being able to compare different quantifying methods, interviewees explained that it was difficult to understand how well different organisations were responding to reducing their carbon emissions in comparison to others:

*‘Microsoft will say that it is carbon neutral by a certain date and it’s aiming towards being carbon negative by another date. But*… *how it calculates its carbon footprint is possibly not identical to the way that Google actually measures its carbon footprint*… *there’s, there’s no way of comparing one to another because they’re not operating across the same metrics.’* (interviewee 4)

One interviewee described how their own company’s policies around carbon quantification might be different to those of other companies: ‘*[this company*’*s] sustainability report, they do not report scope 3. They consider scope three to be somebody else*’*s scope one and two problem*’ (interviewee 13).

Interviewee 19 was concerned that a lack standards meant that double or triple counting could occur in a particular supply chain. They provided the example of a data centre accounting for their emissions through their electricity supply chain:

*‘The data centre operator who is buying electricity from that grid region then takes their scope 1 emission and applies it to my operation. So now I’ve double counted it So then I’m now gonna go to my supplier*… *I say, “ I want to know what your emissions are*,*” So now I’ve counted that CO*_2_ *[carbon dioxide] a third time, right, because it’s been counted by the utility, it’s been counted by my supplier, and now it’s been counted by me*.’

The lack of standards was also perceived to be problematic because standards were viewed as a realisation of the ‘correct’ approach to carbon quantification—they moralised the way in which carbon accounting *should* be completed, and this was seen as something that was much needed in the sector: ‘*lots of different*…*organisations [need to] say*… *“Okay, what, what is fair here?*… *What does good look like?*… *What do we agree that good looks like?*”‘ (interviewee 4). Standards also provided legitimacy for the carbon quantification approach taken. Without standards, and with companies making personal choices about what and how to account for emissions, there was an illegitimacy of carbon accounting and reporting. This meant that any findings lacked meaning outside of those who produced them: as interviewee 12 emphasised, numbers did not become ‘real’ unless they had legitimacy across the sector, and the construction of knowledge only gained legitimacy when the knowledge was standardised:

*‘These things become real when they go across a sector. You know, if you’ve got one company saying, “Well, hey, look. We’re assessed our own practices.” It’s like: “Yeah, okay.” You know, it doesn’t, it doesn’t mean anything unless it’s, unless it’s a kind of common accepted benchmark.’*

The lack of standards was perceived to be related to the newness of the field. Currently, different actors were pushing their own views, and the field had not matured enough to choose a way of seeing and knowing the world. However, moving towards standards was viewed as tricky. Interviewee 11 described the difficulties with trying to reach a consensus in the field because each group of actors was trying to push their own standards as best practice. Interviewee 22, an NGO representative, spoke about their attempts to bring different communities of knowledge together, with only limited progress. In the extract below, they described how they brought together digital technology sector participants from different knowledge communities, academia, and industry to discuss how to standardise calculating carbon emissions for the sector. The meetings, they explained, quickly became dominated by only one or two experts in the specific area under discussion, with those who lack core-set expertise unable to contribute:

*‘I’m working with*… *climate scientists, hardware engineers*… *we’re just not, a little bit not speaking the same language*… *We’ve started a working group.… I would say maybe only three people would be speaking where there’s 30 participants in that call. So it gets very technical, very fast… and then they’ll go into so much detail about the process of something going from A to B and then you know we have a coacher during the call and they’ll say, “well any questions, any comments?” No, because you, we don’t know what we don’t know!’*

## 7 Discussion

Our findings show that, in the digital sector, collecting, agreeing, and acting on quantifiable data during carbon accounting is not easy or straightforward. First, stakeholders deal with complex digital systems, in which it is difficult to disentangle data about the emissions of single systems’ components. Not only are calculations difficult because data are unavailable, incomplete, and/or outdated, but also because decisions must be made on how to draw boundaries across these complex systems, define what aspects and components are considered as valuable and relevant, and which ones are therefore included in the measurement. Second, the methods used to calculate carbon emissions vary across different disciplines. This results in what we referred to as a tension among different research communities, meaning that there is a fragmentation across groups of stakeholders regarding the methodological approaches and implicit assumptions concerning measuring emissions. These groups are sometimes unable to engage in fruitful conversations and struggle to agree on standards or norms that can be used in comparative evaluations. Third, this absence of standards curbs positive action in the field and translates to a lack of shared meaning and sense of guidance for stakeholders, which, in turn, translates to a lack of actionable data. Our findings show a vicious circle from a lack of data that prevents reliable calculations of carbon emissions, that passes through divergent methodologies and approaches, that prevents agreements on common standards, leading to a lack of reliable data which, in turn, makes it difficult to calculate impacts and results in a call for more data.

Our findings can be understood in the context of scholarship that has illustrated how data do not exist objectively in the wild as truths waiting to be discovered but are artefacts that are socially constructed as objects/subjects of knowledge (for example, see [12,13]). This scholarship points to the fact that the uncertainty associated with carbon emissions knowledge production is related to the complex and messy processes involved in materialising data, including the process of framing, selecting, gathering, measuring, operationalising, negotiation, and shaping data, and how these interact with relationships between actors, organisations, and policies [2]. During these processes, personal decisions are often made about what to count and what not to [1,2,5,14]. In line with this scholarship, our findings reveal that carbon emissions counting in the digital sector often start from different assumptions, include individual value-based judgements in their data models, and vary in both scope (what data infrastructure is included in the calculations and what may be left out) and stage of the supply chain measured. The different quantification methods gave rise to different facts and then predictions about the overall carbon emissions attributed to the digital sector, and therefore produced different values associated with the urgency of the sector’s need to reduce carbon emissions [23].

Participants tried to manage this, believing that the imposition of standards could secure this legitimacy in specific methods. They understood that the production of a carbon emission number means little unless others use it, and standards were viewed as a way to achieve this. They also understood that standards are required to have a reliable basis for meaningful change because they facilitate a more collective approach to carbon quantification—the alternative is each community pulling in different directions. In this way, they viewed standards as being able to solve many of the issues and challenges they encountered with carbon quantification.

One way of moving towards standards was to address the tensions among different actors and research communities. However, this was perceived difficult by our participants because of the fragmentation of different research communities, along with the tendency to black-box uncertainties associated with specific methodologies. This is anticipated, given how the broader literature emphasises difficulties with transdisciplinary and multi-sector approaches (for example, see [36–38]). This literature points to how data are viewed through a particular theoretical framework in each discipline, embedded in a particular socio-cultural context within specific institutional power dynamics, and this is how raw data are given meaning [39]. This leads to structural and cultural barriers that lead disciplines to talk past each other, as well as political hierarchies and power asymmetries between individuals, disciplines, and institutions [37,40]. As Scharff and Stone (2022) stress, ‘*what is considered relevant information, what is assumed about how to obtain it, and how one knows when to use it, all vary significantly across disciplines, research programs, and established lines of practice*’ [37]. Our findings show how, with little communication between communities, each community struggled to understand the underlying assumptions baked into the data presented by each other, and understood data in different ways, affecting how they were categorised and counted (for example, see [38,41]).

Furthermore, as our interview findings hinted at, and as the critical literature on standards has long argued (for example, see [42]), while standards are indeed a vital aspect of carbon quantification, standards are themselves social constructions of a particular reality and are deeply political: choosing a standard method is a socio-political process and dependent on which methods (and actors) gain the most prominence through the social and political processes of legitimisation. During standard making, decisions are made about what is counted and what is not. When any standard is implemented, these decisions become accepted, and when these processes and the values embedded within them become normalised and taken for granted in society, and as they become more long-standing and extensive, what is not chosen to be counted becomes invisible in society [1]. Applied to carbon accounting, standards may render some aspects of carbon emissions invisible and/or irrelevant [43,44]; could result in us forgetting to question why and how values come to be expressed quantitively [44]; and/or may reinforce modernist assumptions that place faith in the ability to solve climate change challenges through managing carbon rather than foregrounding the need for broader systemic reforms and/or broader environmental considerations [45,46]. Rebound effects are one example of this, where the focus of quantitively assessing carbon emissions for digital devices, processes, and or services may obscure the fact that increases in energy efficiency (and subsequent reductions in carbon emissions), while perceived to offer environmental advantages, may very likely lead to an increase in digital consumption if systemic reforms are not implemented [45].

This is not to say that standards are not important—of course they are; however, as the community drive calls towards standard making, we need to problematise the belief that the uncertainty around carbon calculations will be resolved once standards are developed, and that these standards will be developed only once we have enough reliable data. This is because uncertainties will always exist and if we wait until they are solved before we develop standards and/or act, this may never come (or may take a long time); in the meantime, we delay taking action to address climate change—which is, of course, the aim of carbon accounting. We argue that perhaps a better way to view the issue is to recognise these uncertainties and the fact that trying to solve them before we act may be untenable. This is not only because carbon emissions’ quantification will never represent an objective and impartial description of reality, and ignoring this can lead to ‘fake precisionism’ [47], but also because it leads to an impasse. In terms of the latter, the community seems to have adopted a particular framing that dooms them to failure: if the aim has been interpreted as needing to arrive at precision and confidence, this will never be achieved. We need to reframe the aim of carbon accounting as one tool in helping us understand the ‘bigger picture’, such that it can act as an indicator for moving in the right direction and/or where to spend effort to reduce emissions. To do so, and to address the lack of agreement between the communities on what to include in the calculations, we can look to the literature on ‘deep uncertainty’ [48].

### Deep Uncertainty

In the early 1990s, Funtowicz and Ravetz recognised that the emergence of complex scientific challenges created by dynamic environmental systems—where ‘*facts [are] uncertain, values in dispute, stakes high and decisions urgent*’—were creating a new phase for the use of science, which they termed ‘post-normal’ science [16,49,50] (1991; p. 138). These authors (1990, p7) claimed that while policy makers had typically expected science to provide straightforward and certain information for their decision-making processes, this was now not possible in climate science because the data included inherent or aleatory uncertainties, which could not be reduced [16,49–51]. As such, science was viewed as faulty or incomplete [52], and inaction suggested as a viable policy option, with reductions in uncertainty being called for through the collection of more data and/or the use of new analysis techniques. However, these and other authors argue(d) that more data often exposes further uncertainties or areas of ignorance [53], with certainty rarely being possible, and so the cycle repeats. Applied to calculating carbon emissions, the need to manage inherent uncertainties in data is required, rather than believing that such uncertainties can be addressed through standards development. This is not least because the fast pace of change in the DT context, as we saw in our findings, means that the development of a standard for calculation would anyway become outdated. Under these uncertainties, decisions are needed to move forward, i.e., decision making under deep uncertainty is required [54].

It is not our intention to suggest a specific method or combination of decision making under deep uncertainty methods because the focus on attention to precision quantification has meant that we have neglected the work needed to create methodologies for dealing more productively with uncertainty, and it is this work that we now need to drive forward. Rather, our aim is to highlight this as a direction that needs to be taken to overcome some of the deep uncertainties we have described, with the goal of drawing policy makers’ and the industry’s attention towards these emerging approaches for making decisions when faced with complex systems, multiple actors, and where standardisation is not feasible and/or is limited in what it is capable of achieving in terms of addressing climate change. Though we do note approaches for dealing with deep uncertainty, such as those described in Marchau et al. (2019) [54], which can provide ways to develop an acceptable or ‘good enough’ way to produce an outcome [55], incorporating elements of adaptability and flexibility so that new knowledge can be included as it becomes available [56]. This could be the development of a simplified, but plausible, framework for carbon quantification using the data that are currently available, alongside logical assumptions. In some areas, this is being carried out, such as the interviewee who was ‘*making stuff up*’ but ‘*doing it intelligently*’. The method can then be updated as data become available or when new technologies are developed. There appears to be a group of people who are prepared to work together, and if they can reach a consensus on this, they could pave the way for others to follow. Crucial to whatever method is taken forward, developed standards must be designed to be flexible for change, with the limitations and assumptions within the standards, as well as *excluded* criteria, being made explicit. Bringing communities together helps with this because it exposes underlying norms, areas of missing data, and/or aspects that remain uncounted. The role of standards then becomes more than just providing an accepted method for counting but also a way of maintaining uncounted elements as exposed, as well as any remaining uncertainties, so that we can move iteratively forward as we gain more data. In essence, standards must not be seen as ‘job done’, but as merely the beginning of addressing issues of deep uncertainty associated with carbon calculations in the digital sector.

## 8 Conclusions

In this paper, we have explored the difficulties with quantifying carbon emissions in the digital sector. We have shown that such challenges have led to uncertainty in the field, with stakeholders calling for more data to address this uncertainty. These calls for more data demonstrate that there will always be uncertainty. We must learn to govern within it through post-normal science approaches. At the same time, we lack suitable frameworks to do so. We call for more research to explore what such a post-normal science framework for carbon accounting in the digital sector could or should look like.

## Supplementary Material

Supplementary Materials

## Figures and Tables

**Table 1:** Self-reported sector of interviewees. Some interviewees had multiple roles.

Self-Reported Sector of Interviewee	Number of Individuals Interviewed
Academic researchers (computer scientists, sustainability experts, social scientists, engineers, societies) (interviewees 1, 2, 6, 7, 8, 11, 16, 17, 23, 24)	10
Industry (commercial, corporate, spin-offs, directors, researchers, alliances, organisations) (interviewees 1, 2, 4, 13, 15, 18, 20, 21)	8
Data centre representative and/or consultant or involved with the sector’s markets (interviewees 2, 9, 10, 13, 21)	5
Policy maker/consultant (funding bodies, organisations associated with standards) (interviewees 3, 5, 12, 14, 19)	5
NGO (interviewee 22)	1

## Data Availability

Data are contained within the article.
