# Supplementary material for: Carbon Accounting in the Digital Industry: The Need to Move towards Decision Making in Uncertainty
Source: Sustainability. Author manuscript; Available in PMC 2024 Sep 16. (PMC7616451; doi:10.3390/su16052017)
Supplement: Supplementary Materials [file EMS198382-supplement-Supplementary_Materials.zip › sustainability-2873594-supplementary.pdf]

Table S1. key-strings used for searching the academic literature on Web of Science.

**Keyword strings**	**Number of retrieved articles**
AB=(environment* sustainab* OR "carbon footprint" OR "environmental footprint" OR "environmental impact" OR "environmental assessment" OR "environmental damage" OR "electricity generation" OR lifecycle OR "carbon emissions" or "greenhouse gases" OR GHG OR "environmental effect" OR "e-waste" OR "end of life" OR "natural resource*" OR biodiversity OR weee OR "circular economy" OR "Paris Agreement" OR "IPCC" OR "climate change" OR "Net-zero" OR "low carbon" OR "energy transition" OR "renewable energy") AND AB=("big data" OR AI OR "artificial intelligence" OR "data cent*" OR ICT OR "information and communication technolog*" OR "cloud computing" OR "internet traffic" OR "machine learning" OR "IOT" or "Internet of things" OR "smart cit*" OR "smart system" OR "smart energy" OR "bitcoin" OR "cryptocurrenc*" or "natural language processing" OR "4th industrial revolution" OR "fourth industrial revolution" OR blockchain)	3,683
AB=("GREEN AI" OR "green ICT")	11
AB=("energy efficiency" OR "energy consumption" OR "energy management") AND AB=sustainab* AND AB=("big data" OR AI OR "artificial intelligence" OR "data cent*" OR ICT OR "information and communication technolog*" OR "cloud computing" OR "internet traffic" OR "machine learning" OR "IOT" or "Internet of things" OR "smart cit*" OR "smart system" OR "smart energy" OR "bitcoin" OR "cryptocurrenc*" or "natural language processing" OR "4th industrial revolution" OR "fourth industrial revolution" OR blockchain)	624
TI and KP=Digital AND AB=(environment* sustainab* OR "carbon footprint" OR "environmental footprint" OR "environmental impact" OR "environmental assessment" OR "environmental damage" OR "electricity generation" OR lifecycle OR "carbon emissions" or "greenhouse gases" OR GHG OR "environmental effect" OR "e-waste" OR "end of life" OR "natural resource*" OR biodiversity OR weee OR "circular economy" OR "Paris Agreement" OR "IPCC" OR "climate change" OR "Net-zero" OR "low carbon" OR "energy transition" OR "renewable energy") [performed as two searches, one title and one keyword searach]	453 (title search) and 146 (keyword search)
